# Setup of an In Vitro Three-Dimensional Stromalized Prostate Cancer Model Using Gelatin Microparticles

**DOI:** 10.1021/acsomega.5c01286

**Published:** 2025-06-02

**Authors:** Giulia Gangarossa, Marta Iozzo, Giulia Mugnaini, Rita Gelli, Luigi Ippolito, Elisa Giannoni, Giuseppina Comito, Massimo Bonini, Paola Chiarugi

**Affiliations:** † Department of Experimental and Clinical Biomedical Sciences, “Mario Serio”, University of Florence, Viale Morgagni 50, 50134 Florence, Italy; ‡ Department of Chemistry “Ugo Schiff” and CSGI, 9300 University of Florence, Via della Lastruccia 3, Sesto Fiorentino, 50019 Florence, Italy

## Abstract

Developing three-dimensional (3D) tumor models that accurately mimic the tumor microenvironment (TME) and its heterogeneity remains a significant challenge in preclinical research. Advancing these models holds the potential to improve the study of cancer pathologies in vitro, while reducing dependence on animal models. To tackle this challenge, in this work, we report on the development of an in vitro 3D stromalized prostate cancer model using gelatin porous microparticles as microscaffolds for cell attachment and growth. Gelatin porous microparticles were prepared by a double emulsion method and cross-linked with a biocompatible cross-linking agent, that is, glyceraldehyde, to prevent dissolution under physiological conditions. Then, we developed a stromalized 3D gelatin-based microscaffold biomimicking the interplay between human prostate cancer (PCa) and stromal cells by coculturing 22Rv1 cells and fibroblasts with gelatin porous microparticles. Overall, our results demonstrate the feasibility of gelatin microscaffolds in reproducing a 3D stromalized model of PCa progression (e.g., metabolic reprogramming), resulting from the tumor–stroma interaction. Thus, these systems represent a valuable platform and an effective tool for the study of cancer progression, such as TME biomimetics, while simultaneously offering a valid alternative to minimize the reliance on animal studies in preclinical research.

## Graphical abstract

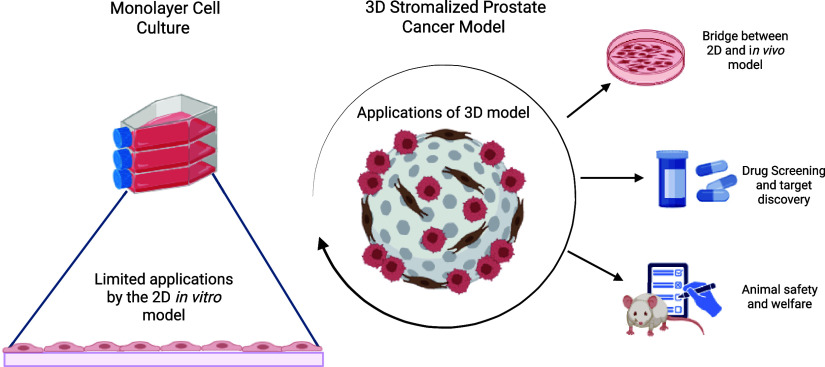

## Introduction

Hydrogels, that is, polymeric networks able to expand throughout their whole volume in aqueous media,^1^ have garnered significant attention in recent decades as scaffolds for supporting the growth of 3D cell cultures, owing to their multiscale structure, high hydrophilicity, and extracellular matrix (ECM) biomimicry.^2,3^ The possibility to fabricate hydrogels using natural polymers, including gelatin,^4^ cellulose,^5^ alginate,^6^ and chitosan,^7^ opened up new horizons and opportunities thanks to their good biocompatibility and easy processability. Conventional (i.e., gas foaming, electrospinning, etc.) and advanced (i.e., 3D and 4D bioprinting) methodologies can be applied for the design and fabrication of hydrogels with appropriate architectures and shapes, including microparticles or microspheres.^8^ The concept of the microcarrier culturing technique developed in 1967 by Van Wezel relies on microparticles made of different polymeric materials that are suspended in the growth medium and act as a support for the growth of anchoring cell lines.^9^ This technology has several advantages, including the possibility to use 3D platforms with a high surface-to-volume ratio and a well-interconnected porosity, which plays an essential role in cells’ survival by diffusion of nutrients and oxygen between the scaffold and the attached cells, promoting their viability.^10,11^ Among the biopolymers which can be used to prepare microscaffolds, gelatin offers a number of advantages^12^ such as the presence of arginine–glycine–aspartic acid (RGD) sequences, improved water solubility and reduced immunogenicity (with respect to its native form, collagen), and the possibility to form thermoresponsive hydrogels, with a gelation point around 30 °C. Additionally, its mechanical and biological properties can be significantly enhanced through cross-linking or other chemical modifications targeting specific functional properties, such as improved stability and resistance to dissolution in physiological conditions.^13^ Chemical cross-linking can be accomplished using a diverse range of cross-linking molecules, including glyceraldehyde, which is highly effective in stabilizing gelatin-based systems and has demonstrated superior biocompatibility compared to standard cross-linkers such as glutaraldehyde.^14^

Due to their biocompatibility and ability to mimic the ECM, gelatin microparticles have showcased their potential as microscaffolds for chondrocyte culture^15^ and human mesenchymal stem cell culture,^16^ and to form spheroids together with adipose-derived stem cells.^17^ Gelatin microscaffolds have recently been used for culturing tumor cells, showing that they support the formation of 3D tumor models, which more accurately replicate in vivo tumor behavior compared to 2D cultures.^18–21^

Thus, 3D structures may also recapitulate the tumor microenvironment (TME),^22,23^ where the interaction among different cell populations underlies the noncell autonomous tumor behavior.^24–26^ In this regard, cancer-associated fibroblasts (CAFs) are the main cellular component of the stromal compartment and are responsible for the ECM deposition and remodeling within a tumor.^27^ Developing 3D tumor models to more closely reflect the tumor heterogeneity, and to minimize the reliance on animal models, faithfully in line with 3R principles (replace, refine, reduce), is a significant challenge in preclinical research.^28–30^ Based on previous research,^31–34^ we developed a stromalized 3D gelatin-based microscaffold biomimicking the interplay between human prostate cancer (PCa) and stromal cells (i.e., fibroblasts) by coculturing 22Rv1 cells and CAFs with gelatin porous microparticles.

## Materials and Methodology

### Materials for Microparticle Preparation

Gelatin from porcine skin (Type A) was purchased from Fluka (48724, Lot #BCBH5042 V, Milan, Italy). TWEEN 80 (Polyoxyethylene 80 sorbitan monooleate, see Figure S1 top in the Supporting Information) was obtained from Merck (Rome, Italy) whereas SPAN 85 (sorbitan trioleate, see Figure S1 bottom in the Supporting Information) was obtained from Bregaglio S.r.l. (Biassono, Italy). Toluene (purity ≥99.8%), ethanol (absolute denatured, ≥99.2% v/v), and acetone (technical grade) were purchased from Carlo Erba (Milan, Italy). D, l-Glyceraldehyde (GAL, purity ≥90% GC) was obtained from Sigma-Aldrich (Milan, Italy). All of the reagents were used without further purification.

### Microparticle Preparation

Gelatin porous microparticles (referred to as SCs) were prepared using a double oil-in-water-in-oil (O/W/O) emulsion method, readapted from the literature.^35^ 0.8 g of gelatin was dissolved in 10 mL of deionized water at 60 °C under stirring, in the presence of 0.3 or 0.9 g of TWEEN 80 (3% w/v or 9% w/v) for obtaining sample SC1 and SC2, respectively. After gelatin dissolution, 5 mL of a toluene solution containing 0.15 g of SPAN 85 (3% w/v) was added keeping the stirring speed at 300 rpm, to form an oil-in-water (O/W) emulsion. Twenty-five mL of toluene was then added to invert the emulsion, leading to an O/W/O emulsion (see Figure S2 in the Supporting Information). In this step, the stirring speed was increased to 500 rpm for sample SC1 and 1000 rpm for sample SC2. After a few minutes, the mixture was transferred to an ice bath, ensuring continuous stirring. When the temperature of the system reached *T* < 10 °C, the stirring was turned off and about 20 mL of cold ethanol was poured into the mixture to extract toluene. Gelatin microparticles were collected by means of filtration under vacuum using a Buchner funnel and thoroughly washed with cold acetone. Particles were dried overnight and then sieved with a 425 μm cutoff metallic sieve, discarding those above 425 μm.

The cross-linking procedure was the same for SC1 and SC2:40 mg of GAL was dissolved in a mixture of acetone (1.32 mL) and water (0.66 mL). The microparticles (200 mg) were then added to the mixture and kept at 5 °C for 24 h. Afterward, the cross-linked microparticles were filtered, washed with acetone, dried overnight under a hood, and finally freeze-dried.

### Optical Microscopy

Optical microscopy was used to determine the size distribution of the prepared SCs. Micrographs were collected with a USB digital microscope (Park Systems). For each sample, about 250 particles were measured using ImageJ software, and radii (with standard deviations) were obtained.

### Field Emission-Scanning Electron Microscopy

The morphology of the scaffolds was studied by means of field emission-scanning electron microscopy (FE-SEM), using a Zeiss ΣIGMA. Microparticles were placed on aluminum stubs by means of conductive tape (no metal coating was applied). Images were collected with an accelerating voltage of 2 kV, using a working distance of about 5 mm and a secondary electron detector.

### Cell Lines

Human PCa cells (22Rv1 - CRL-2505) and human prostate fibroblasts (WMPY-1 - CRL-2854) were obtained from ATCC. All cells were maintained in DMEM (#ECB7501L; Euroclone) supplemented with 10% FBS (#ECS5000L; Euroclone), 2 mmol/L l-glutamine and 1% penicillin/streptomycin. The prostate myofibroblast cell line WPMY-1 was activated using 10 ng/mL of transforming growth factor (TGF)-β in starvation medium for 24 h. As previously reported,^36,37^ fibroblasts activated with TGF-β have similar characteristics to cancer-associated fibroblasts, and they are therefore herein defined as CAFs. All cell lines were maintained at 37 °C and 5% CO_2_ and were routinely tested for *Mycoplasma* contamination with the MycoAlert Mycoplasma Detection kit (#LT07-318; Lonza).

### Preparation of Microparticles for Cell Culture Experiments

The SC2 porous gelatin microparticles were selected for cell culture experiments based on their properties (see Results and Discussion). Microparticles were sterilized by the addition of absolute ethanol (#3221-M; Sigma-Aldrich) at room temperature for 24 h. Subsequently, the SC2 microparticles were washed with buffer solution of phosphate-buffered saline (PBS) and maintained at 4 °C overnight. Then, they were washed twice with PBS by spinning down (300 g, 5 min) and resuspending the pellet with DMEM 2% FBS, and were ready to use for cell culture.

### 3D In Vitro Stromalized Prostate Cancer Cell Model

The establishment of the 3D in vitro PCa cell model was performed in poly-HEMA-coated six-well plates to prevent cell adhesion, by incubating 22Rv1 epithelial PCa cells, alone (1 × 10^5^ cells) or in coculture with stromal cells (2 × 10^5^ WMPY cells, ratio 1:2), with SC2 scaffolds (2 mg/well). The 3D PCa model was cultured in DMEM supplemented with 2% FBS at 37 °C and 5% CO_2_ for 21 days, changing the medium every five days.

### Immunohistochemical (IHC) Analysis

3D PCa cell cultures were collected and fixed in 4% neutral-buffered formalin for 24 h and then included in agarose 1% PBS solution (100 μL for each experimental condition), fixed overnight, processed, and embedded in paraffin (FFPE). Then, hematoxylin/eosin (H&E) and IHC analysis on a sliced section (thickness: 7 μm) obtained from FFPE samples were performed.

As primary antibodies we used: antihuman pan-cytokeratin (#ab234297, Abcam) 1:100, antihuman fibroblast activation protein (FAP, #66562, Cell Signaling) 1:100, antihuman alpha-1 type I collagen (Col1a1, #ab34710, Abcam) 1:100, antihuman monocarboxylate transporter 1 (MCT1, #ab90582 Abcam) 1:500, antihuman fatty acid synthase (FASN, sc-48357, Santa Cruz Biotech) 1:100, antiperilipin-2 (PLIN2, #ab78920, Abcam) 1:100, and anti-H3K27Ac (#8173, Cell Signaling) 1:500. Antibody binding was detected using 3,3′-diaminobenzidine (DAB). Immunohistochemistry was performed using a Leica BOND-MAX automated system (Leica Microsystems). Images were acquired by using a slide scanner (Aperio LV1; Leica Biosystems) and analyzed with ImageScope software ImageScope (RRID:SCR_020993). IHC staining was quantified using ImageJ. Deconvolution was performed using the H DAB algorithm to isolate the DAB staining. The positive area (%) was calculated as the fraction of the stained area over the total analyzed region. All images were processed under the same conditions, and five different areas for each condition were analyzed.

### Analysis of the 3D Structure Area

To analyze the area of the 3D structures, serial sections were obtained from the 22Rv1 and 3D stromalized PCa samples. Images of the sections were acquired using an Aperio LV1 (magnification 2.5X) and analyzed with ImageJ software. The area of the selected structures was measured in each section by defining the region of interest based on morphological criteria.

### Statistical Analysis

Statistical analyses were performed using two-tailed unpaired Student’s *t* test (GraphPad Prism software). Data are presented as mean ± SEM. Statistical significance was considered at * *p* < 0.05, ** *p* < 0.01, *** *p* < 0.001, and **** *p* < 0.0001.

## Results and Discussion

### Characterization of Gelatin Microparticles

Gelatin porous microparticles (SCs) were prepared according to a readapted double oil-in-water-in-oil (O/W/O) emulsion protocol. This procedure relies on the initial formation of an oil-in-water (O/W) emulsion that upon the addition of an excess of toluene undergoes a phase inversion, resulting in an O/W/O emulsion. Following one of our previous studies, the use of different surfactant concentrations and/or stirring speeds in the phase inversion step produces microparticles with marked differences in terms of size, shape, and porosities.^38^ In this work, we specifically focused on two combinations of surfactant concentration and stirring speed: SC1 was prepared with a lower concentration of surfactant (3% w/v) and stirring speed (500 rpm), while both surfactant concentration and stirring speed were increased in SC2 (9% w/v and 1000 rpm, respectively). The samples were then chemically cross-linked using glyceraldehyde (GAL), aiming at preventing the microparticle dissolution in aqueous environments at physiological temperature. As shown in Figure 1A, the cross-linking treatment causes a notable color change in the SCs, shifting from white to yellow. This color change in gelatin-based systems has been previously linked to the formation of an imine covalent bond, known as a Schiff base, between the aldehyde groups of GAL and the ε-amino groups of lysine residues in gelatin, providing direct evidence of cross-linking.^39,40^ Cross-linked scaffolds remained stable in water at 37 °C for over a week, making them suitable for cell culture. The size of the microparticles was determined using optical microscopy (Figure 1B): the average radii and standard deviations are 120 ± 48 μm for SC1 and 103 ± 45 μm for SC2, respectively, as expected from the increase in the stirring speed. The size of the swollen microparticles was also determined, resulting in diameters of 158 ± 39 for SC1 and 154 ± 34 for SC2 (Figure 1C). The size distribution curves of the swollen microparticles are reported in Figure S3. The morphology, specifically the sphericity and porosity, of freeze-dried SCs was investigated by means of scanning electron microscopy. FE-SEM micrographs, depicted in Figure 1D, highlight significant differences in shape and external porosity between the two SCs. Particularly, sample SC2 exhibits a spherical shape with uniformly distributed pores a few micrometers in diameter, consistently with a high surfactant concentration and stirring speed. In contrast, SC1 displays a more irregular shape with larger pores (tens of micrometers in size), alongside smaller pores (a few micrometers in diameter, as shown in the inset in Figure 1D), which could be a consequence of the lower amount of surfactant available for the stabilization of the interfaces.

**Figure 1:**
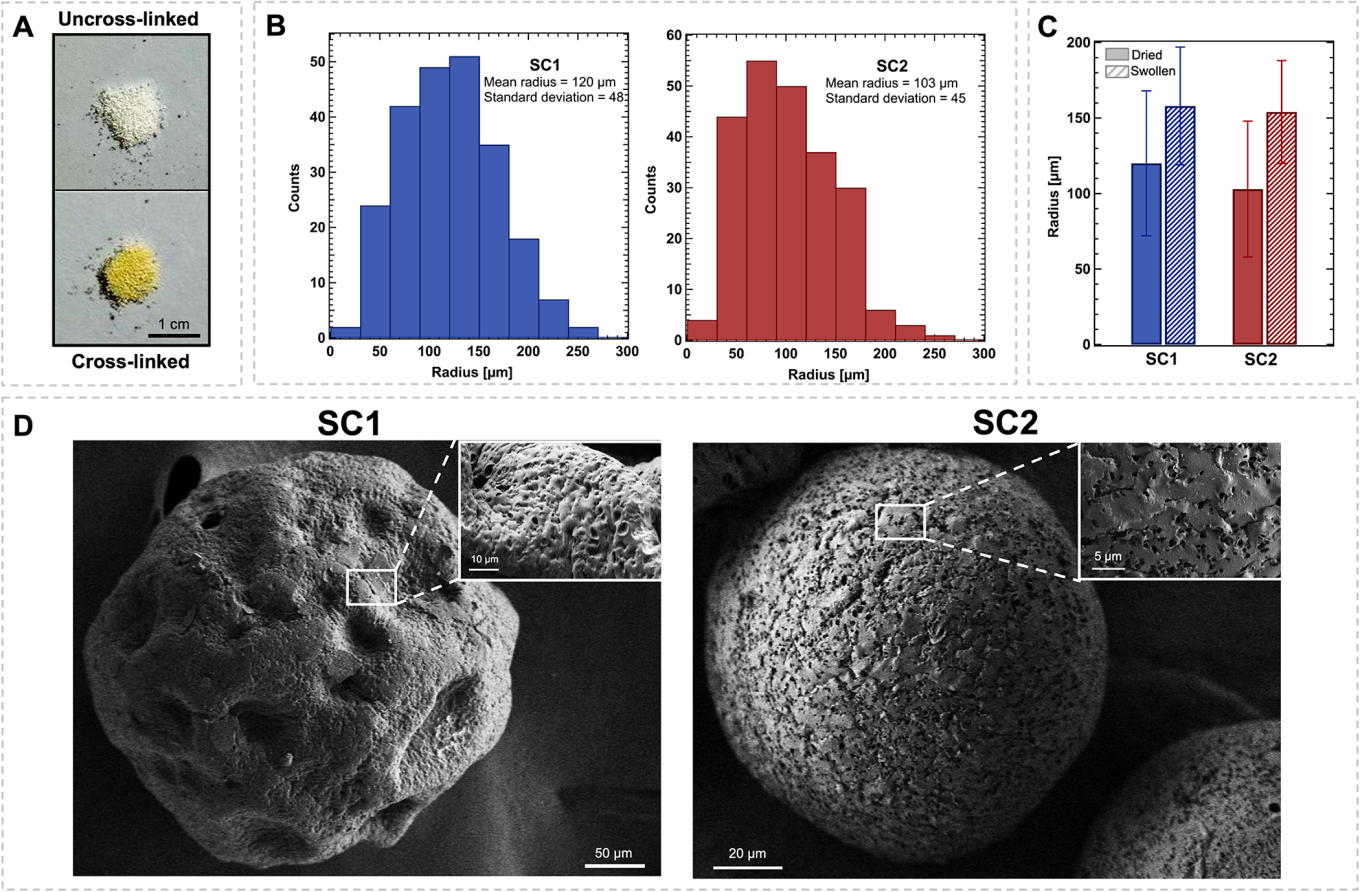
Characterization of gelatin microparticles. (A) Illustrative photograph of gelatin microparticles before (left) and after (right) cross-linking. (B) Size distribution curves obtained from optical microscopy images of SCs radii (average value ± standard deviation); (C) Comparison of the radii (average value ± standard deviation) of dried and swollen microparticles. (D) FE-SEM micrographs of the SC scaffolds.

Based on the obtained results, scaffolds SC2 were selected for the biological experiments, considering that their consistent and reproducible size and shape are expected to provide a more uniform environment for cell adhesion and proliferation, making it easier to model and observe the microscaffold behavior over time.

### Development and Analysis of the 3D Stromalized PCa Cell Model Using SC2 Scaffolds

The SC2 microscaffolds were tested for their ability to generate 3D cellularized structures according to the experimental workflow illustrated in Figure 2. Briefly, following a pilot assay to optimize cells and scaffolds condition, 1 × 10^5^ 22Rv1 cells, either alone or in coculture with 2 × 10^5^ activated WMPY fibroblasts, were seeded onto poly-HEMA coated wells in the presence of 2 mg of microparticles. 3D cultures were monitored daily for a period of 21 days using a bright-field microscope, then collected, and subjected to further analysis.

**Figure 2:**
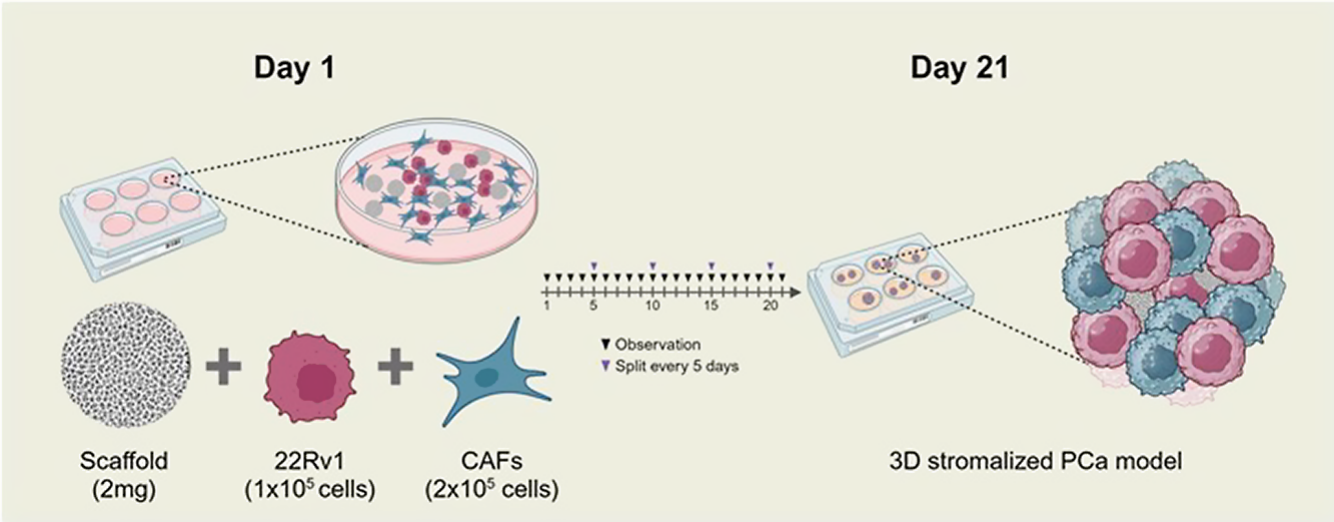
Workflow for developing a 3D stromalized PCa cell model. One × 10^5^ 22Rv1 cells and 2 × 10^5^ CAFs combined with 2 mg of scaffolds (SC2) were maintained in culture for 21 days to obtain a 3D stromalized structure. Created with BioRender.com.

Histological sections stained by H&E of the two compared conditions are shown in Figure 3. In particular, 22Rv1 cells alone were unable to build up a 3D monoculture model (Figure 3A). In contrast, the addition of activated fibroblasts (i.e., CAFs) allows for the formation of a 3D structure, which is prone to be cellularized by tumor cells. This is likely due to the replacement of the microscaffolds with a fibroblast-deposited matrix following an initial gelatin degradation (Figure 3B). Quantitative analysis of the structures revealed a markedly larger area in the 3D stromalized PCa model compared to that of 22Rv1 alone (Figure 3C).

**Figure 3:**
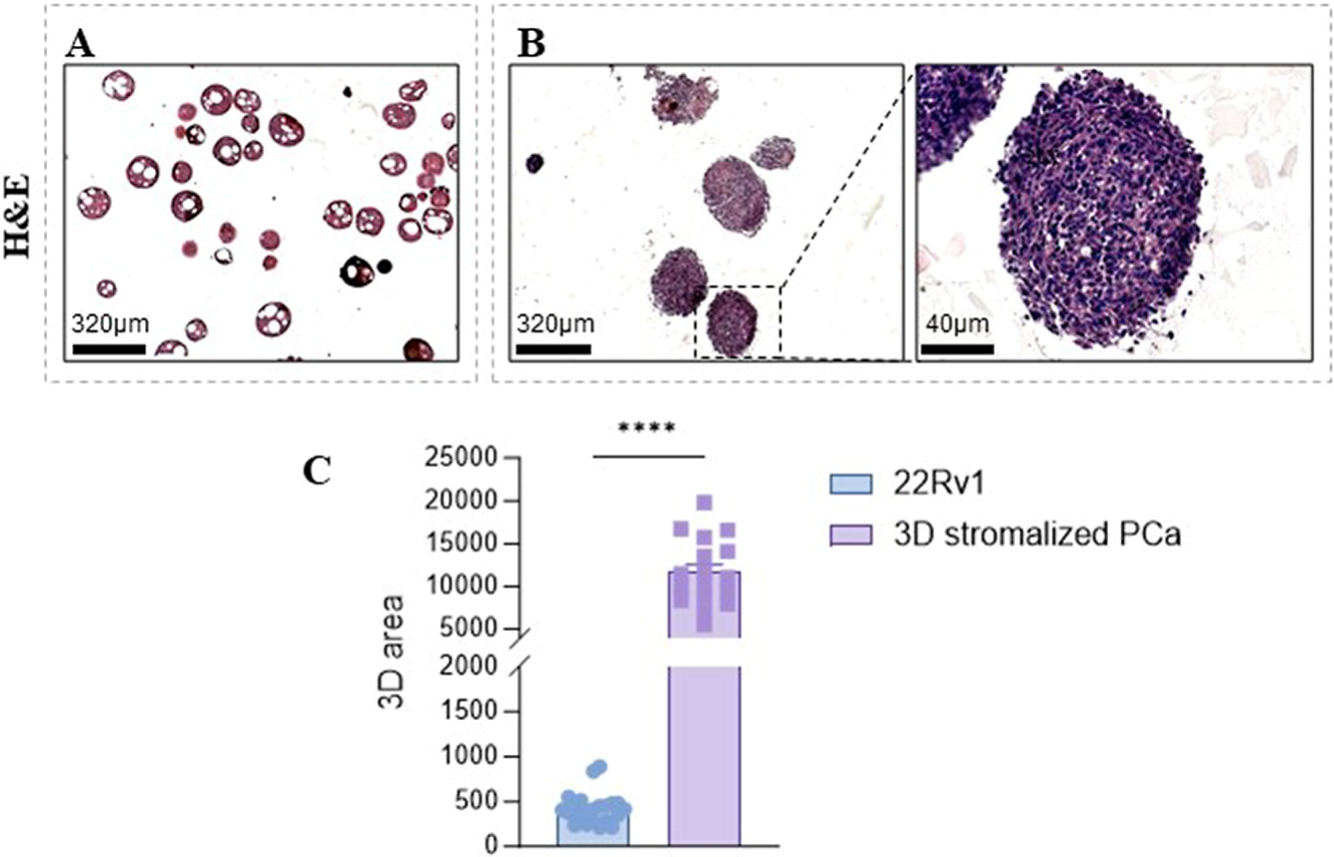
Morphological analysis of the 3D stromalized PCa cell model. (A) Representative H&E images of 22Rv1 cells plated with SC2. Magnification 2.5X, scale bar: 320 μm. (B) Representative H&E images of the 3D stromalized PCa model. Magnification of 2.5X (left) and 20X (right), scale bar: 320 and 40 μm, respectively. (C) Area of target structures measured using ImageJ. Data are reported as AREA with error bars representing means ± SEM of *n* independent serial slides. Two-tailed unpaired Student’s *t* test (C), **** *p* < 0.0001.

To confirm the coexistence in the 3D structures of both 22Rv1 epithelial and stromal cells, we performed an IHC analysis using specific antibodies to selectively recognize both cell types. Particularly, FAP was used as a specific biomarker for CAFs (Figure 4A), while pan-cytokeratin for identifying epithelial tumor cells (Figure 4B). Moreover, Col1a1 immunostaining was used to confirm the deposition of stromal collagen, for which CAFs could be the main contributor (Figure 4C).

**Figure 4:**
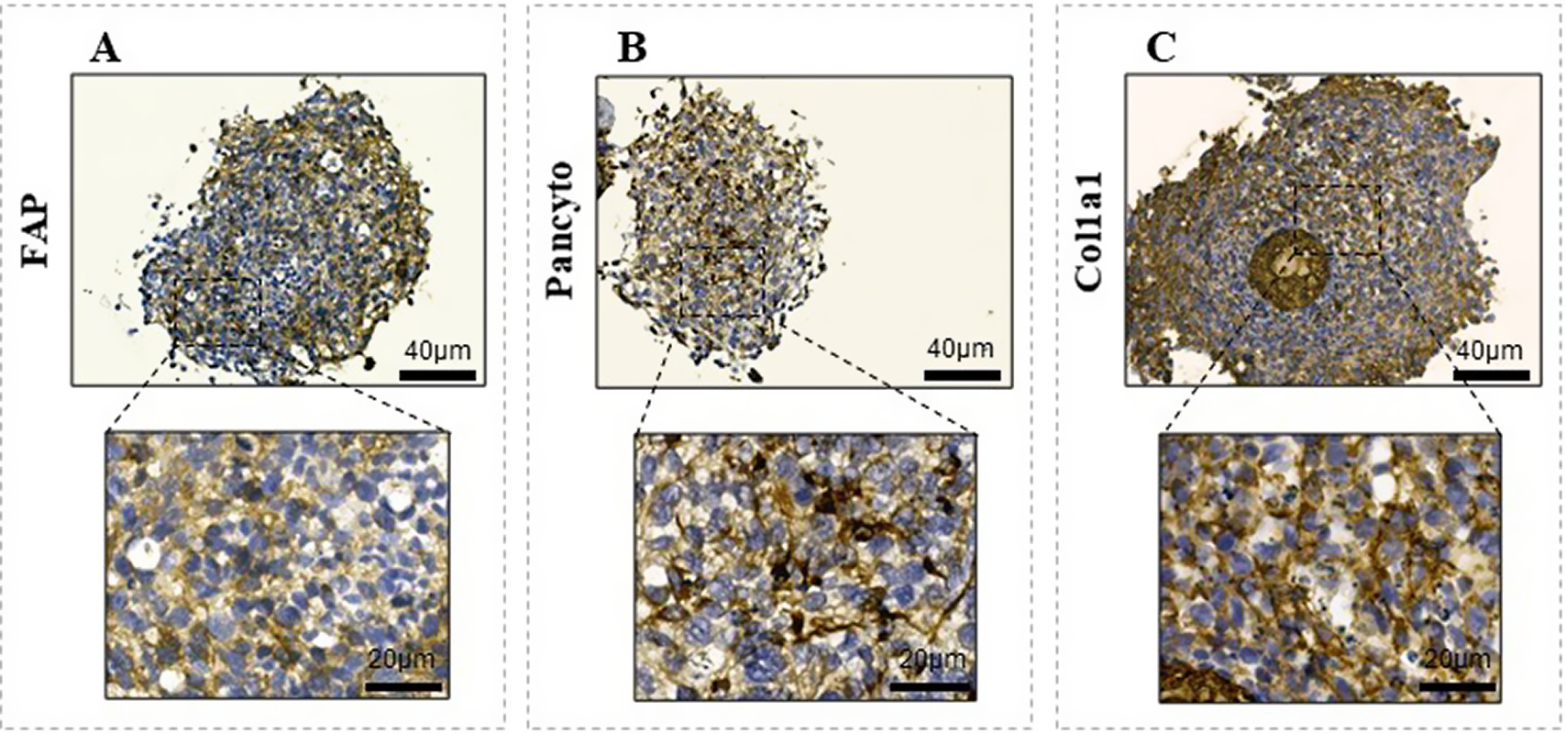
Characterization of the 3D stromalized PCa cell model. (A–C) Representative images of the 3D stromalized PCa model stained for (A) FAP, (B) Pan-cytokeratin, and (C) Col1a1. Magnification 20X and 40X scale bar: 40 and 20 μm, respectively. DAB chromogen was used for all IHC staining.

Next, we reasoned that this 3D structure may resemble PCa tumor-stroma interplay from a metabolic point of view, as previously reported in a 2D cell model describing CAF ability to rewire PCa cell lipid metabolism and histone acetylation.^41^ Interestingly, IHC analysis of the 3D stromalized structures revealed the presence of key markers sustained by CAF conditioning, such as (i) lactate monocarboxylate transporter 1 (MCT1), as readout of lactate transport (Figure 5A); (ii) fatty acid synthase (FASN), a key marker of fatty acid synthesis (Figure 5B); (iii) Perilipin 2 (PLIN2), a lipid droplet-associated protein (Figure 5C); (iv) acetylation of the lysine 27 of histone H3 (H3K27ac) (Figure 5D). The expression of these markers was further supported by quantification, which revealed consistent staining intensities across the 3D stromalized PCa structures, highlighting the active metabolic and epigenetic landscapes induced by CAF conditioning.

**Figure 5:**
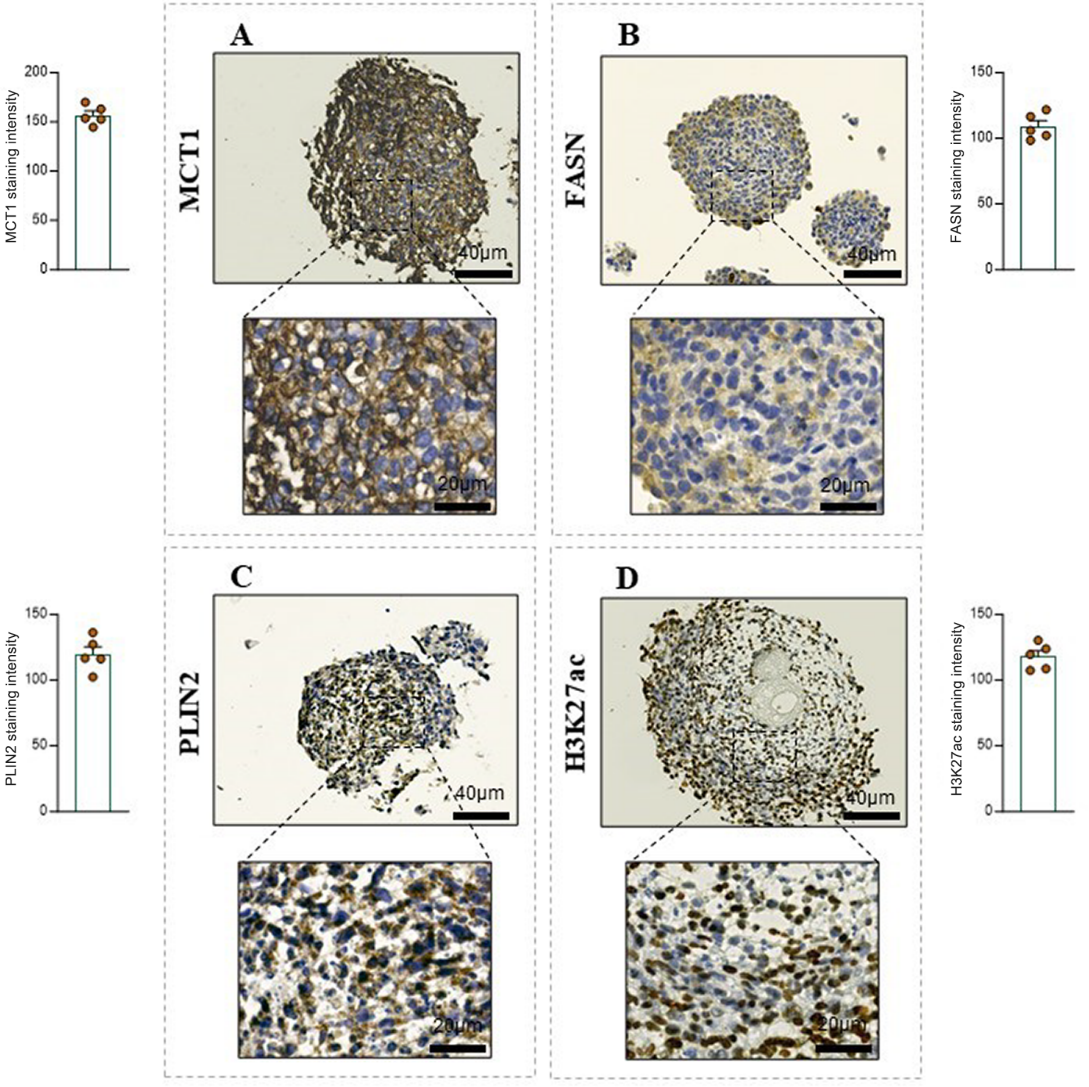
Evaluation of markers involved in cancer prostate metabolic reprogramming. (A–D) Representative images of the 3D stromalized PCa model stained for (A) MCT1, (B) FASN, (C) PLIN2, and (D) H3K27ac. Magnification 20X and 40X, scale bar: 40 and 20 μm, respectively. DAB chromogen was used for all IHC staining. Quantification of the staining intensity for each marker is shown in the corresponding graphs.

Overall, these findings demonstrate the feasibility of SC2 scaffolds in reproducing a 3D stromalized model of PCa progression (e.g., metabolic reprogramming) resulting from the tumor-stroma interaction.

However, additional investigations are needed to optimize their design and to better define the codistribution of specific markers within the different cellular components, in order to make these 3D models more suitable to investigate the reciprocal tumor-stroma metabolic and/or epigenetic adaptations. In addition, to improve the potential of these 3D stromalized models and to enlarge their application also for targeting approaches, different cancer cell lines of prostate origin or derived from different tumor models will to be tested, alone and/or in combination with different microenvironmental components (i.e., immune and endothelial cells).

## Conclusions

This study describes a strategy for developing an in vitro 3D stromalized prostate cancer model taking advantage of porous gelatin microparticles as microscaffolds to recapitulate the TME. The tuning of the synthetic conditions of the double emulsion method allows for obtaining homogeneous spherical particles with a radius of approximately 100 μm and porosities of a few micrometers in diameter. The cross-linking with glyceraldehyde extends the stability of SC microscaffolds at physiological temperature, allowing for cells attachment and survival.

This gelatin scaffold-based system emerges as a promising platform for further research and a valuable tool for studying cancer progression such as TME biomimetics, thereby offering a valid alternative approach to animal studies for preclinical research.

## Supplementary Material



## Notes

Figure S1: Representative molecular structures of TWEEN 80 and SPAN 85; Figure S2: Sketch of the O/W/O emulsion leading to the preparation of porous gelatin microparticles; Figure S3: Size distribution curves of swollen microparticles SC1 and SC2 (PDF)

## Abbreviations

CAFs: cancer-associated fibroblasts
Col1a1: alpha-1 type I collagen
DAB: 3,3′-diaminobenzidine
FAP: fibroblast activation protein
FASN: fatty acid synthase
FE-SEM: field emission-scanning electron microscopy
GAL: glyceraldehyde
H&E: hematoxylin/eosin
MCT1: monocarboxylate transporter 1
O/W: oil-in-water
O/W/O: oil-in-water-in-oil
PBS: phosphate-buffered saline
PCa: prostate cancer
PLIN2: perilipin-2
SC: gelatin porous microparticle
TGF-β: transforming growth factor β
TME: tumor microenvironment
