# Supplementary material for: Setup of an In Vitro Three-Dimensional Stromalized Prostate Cancer Model Using Gelatin Microparticles
Source: ACS Omega. 2025 Jun 2;10(22):23121–8. doi: 10.1021/acsomega.5c01286 (PMC12163836; doi:10.1021/acsomega.5c01286)
Supplement: Supplementary file 1 [file ao5c01286_si_001.pdf]

Setup of an *in vitro* 3D stromalized prostate cancer model using gelatin microparticles

Giulia Gangarossa [1], *Marta Iozzo* [1], *Giulia Mugnaini* [2], *Rita Gelli* [2], *Luigi Ippolito* [1], *Elisa Giannoni* [1], *Giuseppina Comito* [1], *Massimo Bonini* [2], *Paola Chiarugi* [1*]

[1] Department of Experimental and Clinical Biomedical Sciences, "Mario Serio". University of Florence, Viale Morgagni 50, 50134, Florence, Italy

[2] Department of Chemistry "Ugo Schiff" and CSGI, University of Florence, Via della Lastruccia 3, 50019, Sesto Fiorentino, Florence, Italy

[*] Corresponding author: P. Chiarugi, paola.chiarugi@unifi.it

SUPPORTING INFORMATION.

FIGURE S1

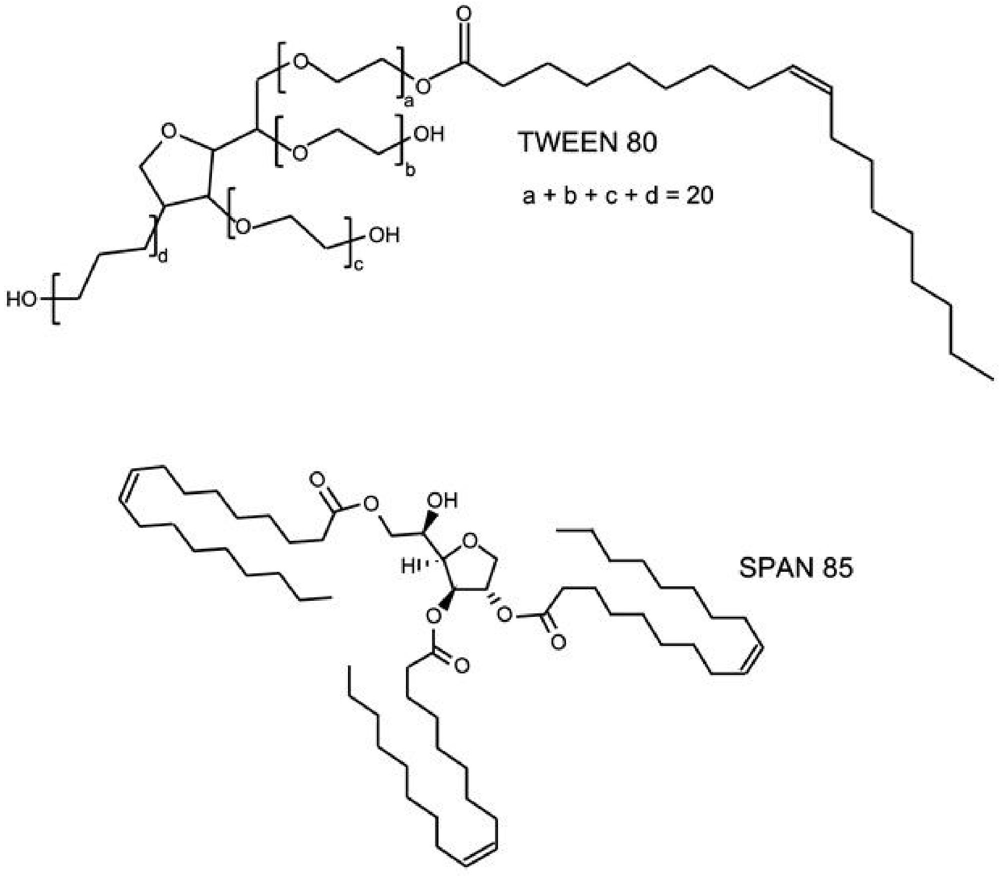

Figure S1. Representative molecular structures of TWEEN 80 (top) and SPAN 85 (bottom).

FIGURE S2

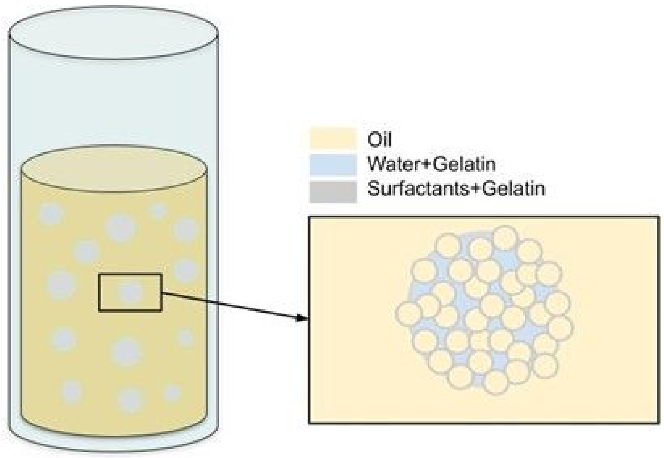

Figure S2. Sketch of the O/W/O emulsion leading to the preparation of porous gelatin microparticles.

FIGURE S3

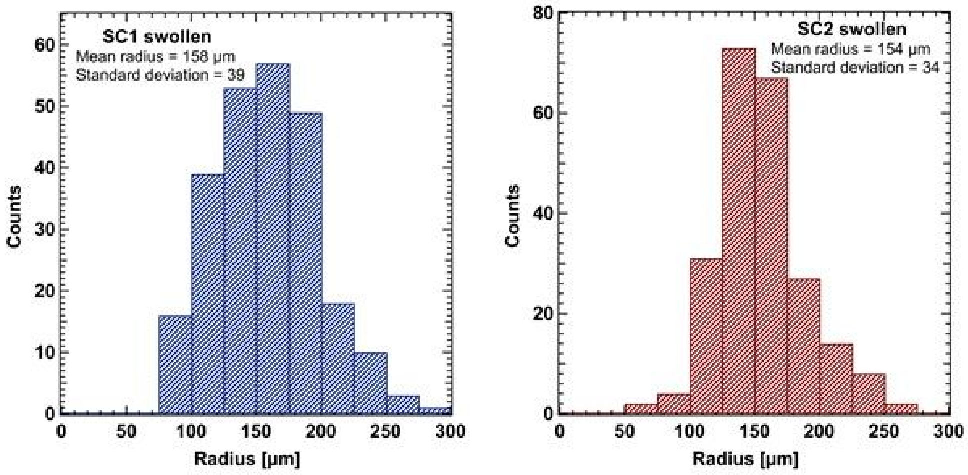

Figure S3. Size distribution curves of swollen microparticles SC1 and SC2.
